# Patterns and determinants of adherence to resistance and endurance training during cancer treatment in the Phys-Can RCT

**DOI:** 10.1186/s13102-022-00548-5

**Published:** 2022-08-13

**Authors:** Hannah L. Brooke, Anne-Sophie Mazzoni, Laurien M. Buffart, Sveinung Berntsen, Karin Nordin, Ingrid Demmelmaier

**Affiliations:** 1 Department of Surgical Sciences, Uppsala University, Uppsala, Sweden; 2 Department of Public Health and Caring Sciences, Uppsala University, Uppsala, Sweden; 3 Department of Physiology, Radboud University Medical Center, Radboud Institute for Health Sciences, Nijmegen, The Netherlands; 4 Department of Sport Science and Physical Education, University of Agder, Kristiansand, Norway

**Keywords:** Adherence, Resistance training, Endurance training, Exercise, Cancer, FITT principles, Determinants, Patterns

## Abstract

**Background:**

Knowledge regarding adherence is necessary to improve the specificity of exercise interventions during cancer treatment. We aimed to determine adherence to resistance and endurance training interventions in parallel; identify subgroups with similar adherence characteristics; and examine determinants of these subgroups.

**Methods:**

In the Phys-Can randomised controlled trial, participants (n = 577, 81% women, mean(SD) age 59(12) years, and 50% with BMI ≥ 25 kg/m^2^) starting (neo-) adjuvant treatment for breast, colorectal or prostate cancer were randomized to 6-month of high (HI) or low-to-moderate intensity (LMI) supervised, group-based resistance training and individual home-based endurance training, with or without behavior change support. Adherence was calculated as performed exercise volume as a proportion of prescribed exercise volume (0–100%), overall (HI and LMI groups) and for frequency, intensity, type and time (FITT principles) (HI group). Adherence to resistance training was plotted against adherence to endurance training overall and for each FITT principle. K-means cluster analysis was used to identify subgroups with similar adherence characteristics. Potential determinants of subgroup membership were examined using multinomial logistic regression.

**Results:**

We found a positive curvilinear correlation between adherence to resistance and endurance training overall. A similar correlation was seen for adherence to frequency of resistance vs. endurance training in the HI group. In the HI group, adherence to resistance training intensity and time was > 80% for almost all participants. For endurance training adherence ranged from 0 to 100% for each of the FITT principles. Three clusters were identified, representing low, mixed, and high adherence to resistance and endurance training overall. Participants with higher age (Relative risk ratio [95% CI]; LMI: 0.86[0.77–0.96], HI: 0.83[0.74–0.93]), no behaviour change support (LMI: 0.11[0.02–0.56], HI: 0.20[0.05–0.85]), higher cardiorespiratory fitness (LMI: 0.81[0.69–0.94], HI: 0.80[0.69–0.92]), more fatigue (according to the reduced activity subscale of the MFI questionnaire) (LMI: 0.48[0.31–0.73], HI: 0.69[0.52–0.93]) or higher quality of life (LMI: 0.95[0.90–1.00], HI: 0.93[0.88–0.98]) were less likely to be in the low than the high adherence cluster whether randomised to LMI or HI training. Other determinants were specific to those randomised to LMI or HI training.

**Conclusions:**

In an exercise intervention during cancer treatment, adherence to resistance and endurance training were positively correlated. Personalisation of interventions and additional support for some subgroups of participants may improve adherence.

*Trial registration* NCT02473003 (clinicaltrials.gov, Registered 16/06/2015).

## Background

Exercise interventions in individuals diagnosed with cancer are safe and help reduce side effects, such as cancer-related fatigue [1, 2], and increase health-related quality of life [3]. As such, international exercise guidelines for cancer survivors recommend at least 3 weekly sessions of 30 min moderate-intensity aerobic training, and/or at least 2 weekly sessions of resistance training [1]. However, to further improve the specificity of recommendations for exercise during cancer treatment, more evidence regarding the optimal ‘exercise prescription’ in terms of exercise frequency, intensity, time, and type, (i.e. the FITT principles) is required [1]. To support this goal and optimise exercise interventions it is vital to understand adherence to exercise interventions in detail, ideally, for each of the FITT principles [4].

In 15 high-quality studies of exercise interventions during and after multimodal cancer treatment adherence rates ranged from 60 to 90% [5]. However, in these studies adherence was largely synonymous with attendance, and did not consider whether the intervention was performed as prescribed in terms of intensity, time and type. Indeed, most exercise-oncology trials do not report adherence to all components of the prescribed intervention [4, 6].

In studies that have aimed to examine adherence to exercise interventions during oncological cancer treatment in more detail, adherence has been presented as an average for resistance exercise and/or endurance exercise, either overall or according to the FITT principles [7, 8]. However, in interventions that include both resistance and endurance training it is important to understand if individual participants are equally adherent to both types of training. The interpretation of results and the implications for future studies may vary substantially if a large proportion of participants within a study are more adherent to resistance training than endurance training or vice versa. Such possibilities may be concealed when examining average adherence levels for the entire sample, for example if two subgroups with different patterns of adherence balance each other out on average.

Comparing determinants of adherence to exercise interventions between studies is challenging due to the wide variety in the type, intensity and duration of exercise interventions that have been delivered, and the different populations and cancer types that have been studied. Nonetheless, baseline physical fitness and previous exercise history are among the few demographic and clinical characteristics have been associated with adherence to exercise interventions in cancer survivors during treatment in several studies [5, 9]. However, these studies focus on adherence to the intervention overall and few have had the opportunity to separate determinants of adherence to low-to-moderate intensity exercise from determinants of adherence to high intensity exercise.

A better understanding of adherence patterns and the determinants of adherence at a detailed level will help optimise future interventions and better target exercise interventions to the capabilities of patient groups. This will enable us to identify particular subgroups of the population who may require additional support or interventions to be adapted to their needs. Thus allowing adequately prescribed interventions and realistic goals to be set, which is important to motivate behaviour change.

We hypothesised that there may be subgroups of participants with high adherence to resistance training but low adherence to endurance training, or vice versa*,* and that we may be able to identify subgroups of participants with similar adherence characteristics. Furthermore, we hypothesised that some demographic and clinical characteristics may be associated with adherence to resistance and endurance training, highlighting subgroups of participants who may require additional support or interventions to be adapted to their needs. The aims of this study were to (1) describe patterns of adherence to resistance and endurance training in parallel, overall and according to the FITT principles; (2) identify subgroups of individuals with similar adherence characteristics; (3) examine demographic and clinical determinants of adherence to resistance and endurance training.

## Methods

### Study design and participants

**Fig. 1:**
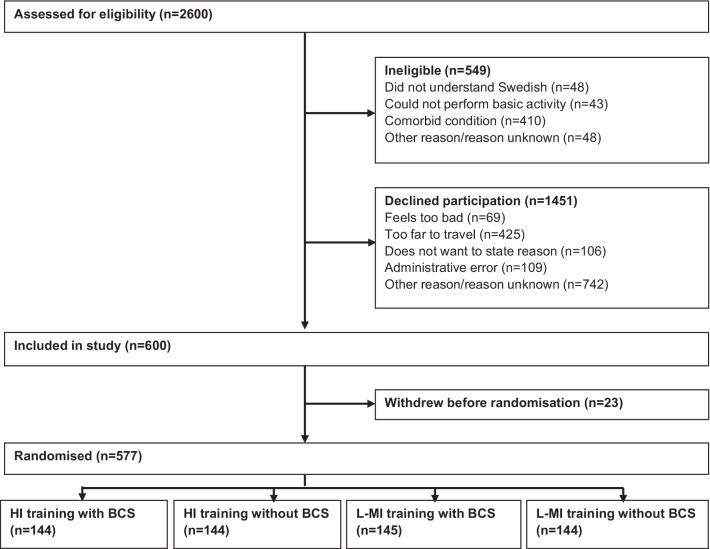
CONSORT flow chart showing flow of participants through to randomisation in the Phys-Can study. HI, high intensity; L-MI, low-to-moderate intensity, BCS; behaviour change support

The Physical training and Cancer (Phys-Can) study is a multicentre, 2 × 2 factorial design, randomised controlled trial (RCT) (NCT02473003, Registered 16/06/2014,) previously described in depth [10, 11]. Participants, were recruited from Uppsala, Lund and Linköping University hospitals (March 2015–April 2018). Eligibility was assessed by an oncologist. The eligibility criteria stipulated that participants were > 18 years old, literate in Swedish and recently diagnosed with curable breast (women only), prostate or colorectal cancer, scheduled to begin (neo-)adjuvant chemotherapy, radiotherapy, and/or endocrine therapy. Exclusion criteria were stage IIIb–IV breast cancer, inability to perform basic activities of daily living, cognitive disorders, severe psychiatric disease, or other disabling conditions that might contraindicate high-intensity exercise (e.g., severe heart failure, severe chronic obstructive pulmonary disease, or orthopaedic conditions), treatment for an additional ongoing malignant disease, BMI < 18.5 kg/m^2^ or pregnancy. In total, 2051 eligible individuals were identified, of which 600 agreed to participate (see CONSORT flow chart, Fig. 1). Twenty-three participants withdrew from the study before randomization, so in total, 577 participants were randomized (computer generated random allocation sequence concealed from all research staff) to high or low-to-moderate intensity exercise, with or without additional behaviour change support. All participants provided written informed consent. The study was approved by the Regional Ethical Review Board in Uppsala (Dnr 2014/249). The research was performed in accordance with the relevant guidelines including the Declaration of Helsinki.

### Description of intervention

The 6-month intervention, previously described in detail [7, 11], was conducted between March 2015 and November 2018, during each patient’s treatment period. In brief, it consisted of supervised, group-based resistance training at public gyms twice per week and individual home-based endurance training. Resistance training for participants randomised to high intensity exercise was 3 sets of 6 exercises at an intensity of 6 repetitions maximum (RM) (first weekly session) or 10 RM (second weekly session). Participants continued to failure in the last set of each exercise. Resistance training for participants randomised to low-to-moderate intensity exercise was 3 sets of 6 exercises at 50% of maximal muscle strength, with 12 repetitions per set (first weekly session) and 20 repetitions per set (second weekly session). According to the protocol, the total volume of resistance training was therefore the same for both exercise intensities. The prescribed endurance training in the high intensity groups was interval training twice per week, composed of two minutes of exercise (e.g. running, cycling or walking uphill) at 80–90% of heart rate reserve (based on a cardiopulmonary treadmill test to exhaustion performed before randomisation) followed by two minutes of active rest. Participants started with five intervals and an additional interval was added every fourth week to a maximum of 10 intervals. The endurance training in the low-to-moderate intensity groups consisted of at least 150 weekly minutes of endurance training (e.g. walking or biking) in bouts of at least 10 min at 40–50% of heart rate reserve. Additional behaviour change support such as goal-setting, planning and self-monitoring was delivered face-to-face on nine occasions for participants randomised to receive this additional support. However, we previously found no effect of behaviour change support on exercise adherence [7] so results are stratified by training intensity only.

### Measures of adherence

For all individuals, adherence to the resistance training and endurance training components of the intervention were calculated overall and each of FITT principles as performed exercise as a proportion of prescribed exercise (Table 1) [7].

**Table 1:** Calculations for training adherence overall and for each of the FITT principles. Adapted from Mazzoni et al. [7]

	Resistance training	Endurance training
Overall	Performed reps × sets × weight/prescribed reps × sets × weight	Performed minutes at prescribed intensity/prescribed minutes
Frequency	n attended sessions/n prescribed sessions	n performed sessions/n prescribed sessions*
Intensity	Performed weight/prescribed weight during performed sessions	n of performed intervals where the target heart rate zone was met/n of prescribed intervals during performed sessions*
Time	Performed sets*repetitions/prescribed sets*repetitions during performed sessions	Duration of performed intervals/prescribed duration of intervals during performed sessions*
Type	n sessions where all prescribed exercises were performed/ n performed sessions	n performed interval training sessions/n performed sessions*

*Only possible to calculate for the high intensity training groups because the exercise prescription for endurance training in the low-to-moderate groups did not include frequency, as it was considered unfeasible to prescribe number of bouts

Adherence to resistance training was based on checklists of attendance; results from 6 and 10 RM tests reported by coaches; and resistance training logs including repetitions completed, sets completed, and weight achieved for each exercise, recorded by the participants at each training session. Adherence to endurance training was primarily based on objective data from heart rate monitors that participants wore during each endurance training session. This was complemented by self-reported training logs to help ensure completeness of the data. Adherence to resistance and endurance training overall was calculated for participants who trained at both and low-to-moderate and high intensity. However, it was only possible to calculate adherence to each of the FITT principles for those participating in the high intensity training groups. This was because the exercise prescription for endurance training in the low-to-moderate intensity training groups did not include frequency, as it was considered unfeasible to prescribe the number of bouts and we focused on weekly recommendations, as in current physical activity guidelines.

### Potential determinants

Potential determinants of intervention adherence were measured before randomisation to the four intervention arms except for information about treatment with chemotherapy. Treatment with chemotherapy was extracted from medical records at the end of the intervention based on the type of treatment received.

The characteristics to examine were decided a priori based on previous knowledge and availability of data. Potential determinants of interest were participant age (continuous variable), type of cancer (breast, colorectal, prostate), study centre (Linköping, Lund, Uppsala), intervention group (with or without behaviour change support), received chemotherapy according to medical records post-intervention (yes vs. no), self-reported comorbidity at baseline (yes vs. no), baseline cardiorespiratory fitness quantified as maximal oxygen uptake during walking/running to exhaustion using a modified Balke protocol [12] (continuous variable), baseline stage of change for endurance training and for resistance training (based on Exercise Stage Assessment Instrument category 5, i.e. Maintenance stage, physically active longer than 6 months, vs. categories 1–4, i.e. Pre-contemplation stage-Action stage), five dimensions of fatigue assessed at baseline with the Multidimensional Fatigue Inventory (MFI) [13] (continuous variables), and function and symptom scales from the European Organization for Research and Treatment of Cancer (EORTC) core quality of life questionnaire (QLQ-C30) [14] (continuous variables). However, the EORTC C30 symptom scales for financial insecurity was not included because the training sessions were provided without cost for the participants.

### Statistical analysis

Scatter plots of overall adherence to resistance training against overall adherence to endurance training are presented separately for participants randomised to low-to-moderate intensity training and those randomised to high intensity training. For participants randomised to high intensity training, scatter plots of adherence to resistance training against adherence to endurance training are presented for training frequency, intensity, time and type. The curvilinear correlation between adherence to the resistance training and endurance training was assessed based on Spearman’s ρ. Cubic splines were fitted with 5 cross-median knots. This allow us to illustrate the relationship between overall adherence to resistance training and overall adherence to endurance training, for participants randomised to low-to-moderate intensity training and those randomised to high intensity training. Cubic spline fitted with 5 cross-median knots were also used to show the relationship between adherence to prescribed frequency of resistance training and adherence to prescribed frequency of endurance training, for participants randomised to high intensity training.

K-means cluster analysis was used to divide the sample into groups with similar adherence characteristics. Clusters were based on overall adherence to resistance and endurance training for participants randomised to low-to-moderate intensity training and those randomised to high intensity training separately. Cluster analyses excluded participants with 0% adherence to both endurance and resistance training. To determine the most appropriate number of groups to specify we visually inspected plots of the within sum of squares and its logarithm, the η2 coefficient, and the proportional reduction of error coefficient for 0–20 clusters [15].

Potential determinants of membership to the clusters, based on overall adherence to endurance and resistance training for participants randomised to low-to-moderate intensity training and those randomised to high intensity training separately, were examined using multinomial logistic regression. All potential determinants were included in a mutually adjusted model. The model was fitted for individuals with complete data on all co-variates.

Analyses were run in Stata version 15.0.

## Results

### Participants

In total, 577 patients were randomised to high or low-to-moderate intensity exercise, with or without additional behaviour change support. Participant characteristics were similar for those randomised to high vs. low-to-moderate intensity exercise (Table 2). The majority of participants were highly educated (58% with tertiary education) normal weight (50%) women (80%) with breast cancer, treated with chemotherapy (53%). Most participants were in a ‘preparation’ stage of change or higher, indicating that they did not perceive themselves as especially physically active but that they were determined to increase their activity level over the following 6 months.

**Table 2:** Baseline characteristics of participants in the Phys-Can Study

	Randomised to 6 months of	
	Low-moderate intensity training (n = 289)	High intensity training (n = 288)
Age (years) mean (SD)	59 (12)	59 (12)
Sex n (%)		
Male	55 (19)	57 (20)
Female	234 (81)	231 (80)
Education n (%)		
Primary	32 (11)	30 (11)
Secondary	59 (21)	77 (28)
Tertiary	173 (62)	163 (58)
Other	15 (5)	9 (3)
Diagnosis n (%)		
Breast cancer	229 (79)	228 (79)
Colorectal cancer	12 (4)	11 (4)
Prostate cancer	48 (17)	49 (17)
Weight status n (%)		
Underweight/normal weight (BMI < 25 kg/m^2^)	135 (50)	132 (49)
Pre-obese (BMI ≥ 25 kg/m^2^ < 30 kg/m^2^)	98 (36)	90 (33)
Obese (BMI ≥ 30 kg/m^2^)	39 (14)	48 (18)
Treated with chemotherapy n (%)	157 (55)	151 (52)
Comorbidities n (%)		
None	109 (39)	113 (42)
One or more	170 (61)	157 (58)
Exercise stage of change* median (IQR)		
Cardiovascular training	3 (3–5)	3 (3–5)
Strength training	3 (2–3)	3 (2–4)
VO_2_ max (mL/kg/min) mean (SD)	30.1 (7.1)	30.7 (7.1)
MVPA (h/day) mean (SD)	1.2 (0.8)	1.2 (0.9)

SD, standard deviation; BMI, Body Mass Index; IQR, Interquartile range; VO_2_ max, maximal oxygen uptake; MVPA, moderate-to-vigorous intensity physical activity as measured by 7 day Sensewear accelerometer

*Exercise stage assessment instrument categories 1–5 with 1 = pre-contemplation stage and 5 = maintenance stage, physically active longer than 6 months. n varies due to missing data, % is of those with data available

### Adherence patterns

**Fig. 2:**
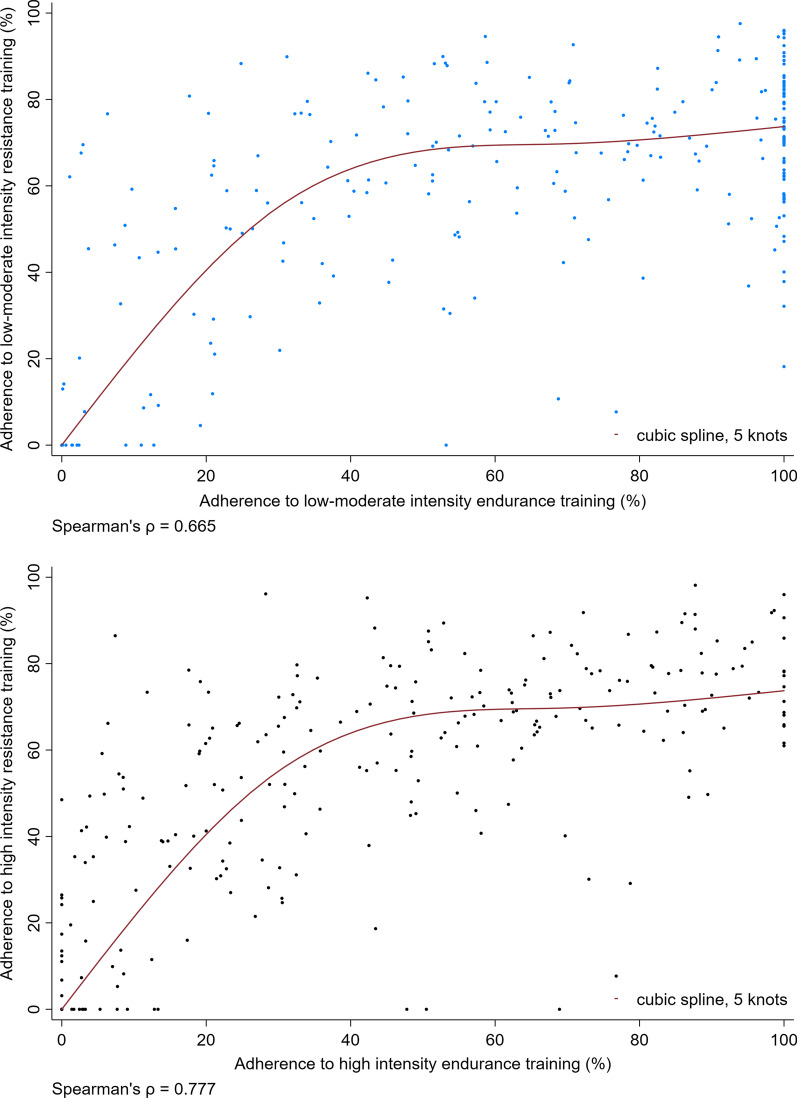
The correlation between adherence to resistance training overall and adherence to endurance training overall. NB. Cubic splines were fitted with 5 cross-median knots to illustrate the relationship between overall adherence to resistance training and overall adherence to endurance training for each training intensity group

Adherence to resistance training overall had a positive curvilinear correlation with adherence to endurance training overall for participants randomised to low-to-moderate intensity training and those randomised to high intensity training (Spearman’s ρ: 0.665 low-to-moderate intensity training; ρ: 0.777 high intensity training) (Fig. 2). Visually inspecting Fig. 2 showed that for participants with less than 30% adherence to endurance training, adherence to resistance training was twice as high as adherence to endurance training among participants randomised to low-to-moderate intensity training and those randomised to high intensity training. However, at both training intensities there was a plateau in adherence to resistance training between 65 and 75% and very few participants achieved more than 90% adherence to resistance training. In contrast, 100% adherence to endurance training was achieved by 23% of participants in the low-to-moderate intensity training groups and 5% of participants in the high-intensity training groups. Among the individuals achieving 100% adherence to endurance training, there was a wide distribution in adherence to resistance training, particularly among participants in the low-to-moderate intensity training groups.

**Fig. 3:**
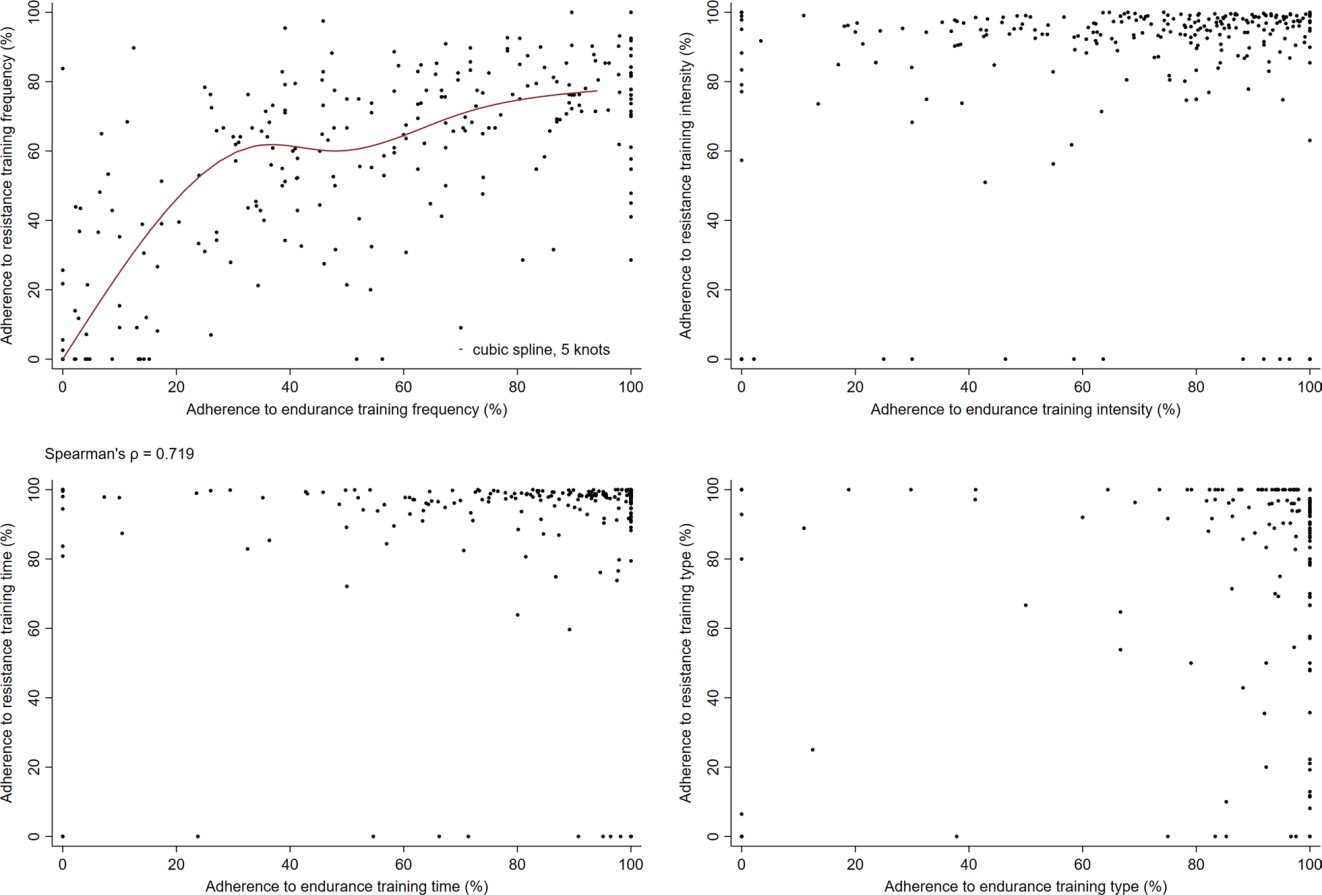
Correlation between adherence to resistance training and endurance training frequency FITT principles. Figure shows the correlation between adherence to resistance training and endurance training frequency (**A**), intensity (**B**), time (**C**), and type (**D**) (FITT principles) for participants randomised to high intensity training (n = 288). NB. Cubic splines were fitted with 5 cross-median knots to illustrate the relationship between adherence to prescribed frequency of resistance training and adherence to prescribed frequency of endurance training

Among participants randomised to high intensity training, adherence to prescribed *frequency* of resistance training had a positive curvilinear correlation with adherence to prescribed *frequency* of endurance training, similar to the pattern for overall adherence (Fig. 3A). While adherence to prescribed *intensity* and *time* for endurance training was 0–100%, for resistance training adherence to these FITT principles was above 80% for almost all participants (Fig. 3B, C). Adherence to training *type* was high for both resistance and endurance training (Fig. 3D). However, there were subsets of participants with 100% adherence to endurance training type but with a range of adherence to resistance training type from 0–100% and vice versa.

**Fig. 4:**
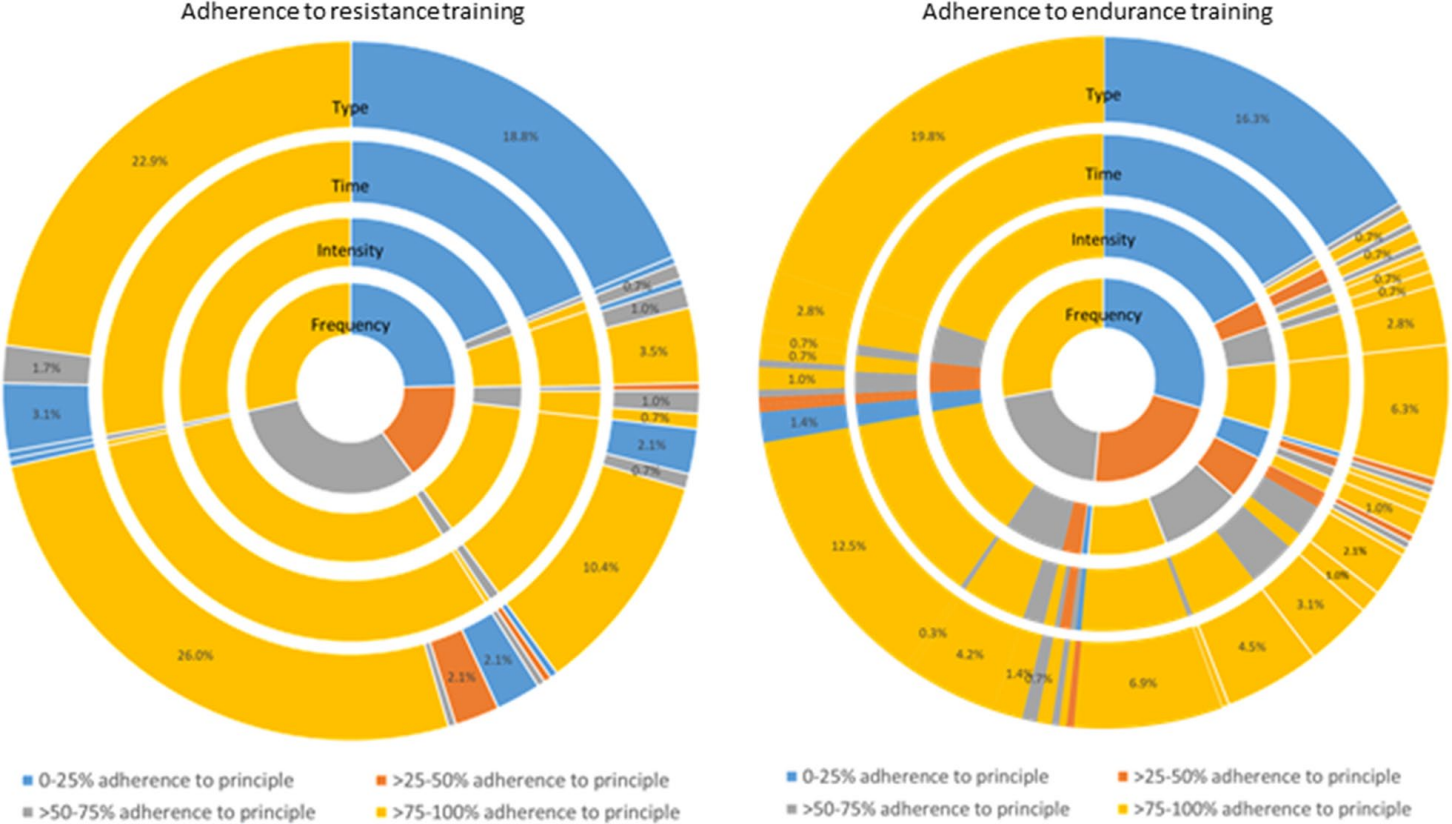
Patterns of adherence to resistance and endurance training frequency, intensity, time, and type for participants randomised to high intensity training (n = 288). NB. Each’slice’ of the figure represents the proportion of participants with a specific combination of adherence levels for the four FITT principles, for example, 18.8% of participants had 0–25% adherence to all four FITT principles for resistance training, while 3.5% of participants had 0–25% adherence to the prescribed resistance training frequency but 75–100% adherence to the prescribed resistance training intensity, time and type. Blue segments represent 0–25% adherence, orange segments represent > 25–50% adherence, grey segments represent > 50–75% adherence, and yellow segments represent > 75–100% adherence

For resistance training in the high intensity training groups, participants were approximately equally distributed across the levels of adherence to frequency (Fig. 4). Within each level of adherence to frequency > 25%, the large majority were 75–100% adherent to intensity, time and type; nonetheless, there was a small subset (2–3%) who were only 0–25% adherent to type despite being 75–100% adherent to intensity and time. For endurance training, compared to resistance training, there were many more different combinations of adherence to each of the four FITT principles. For endurance training, within each category of adherence to frequency there was a substantial proportion of individuals with < 75% adherence to intensity. However, within each level of adherence to frequency > 25%, the large majority were 75–100% adherent to endurance training type.

### Adherence characteristics (cluster analysis)

**Fig. 5:**
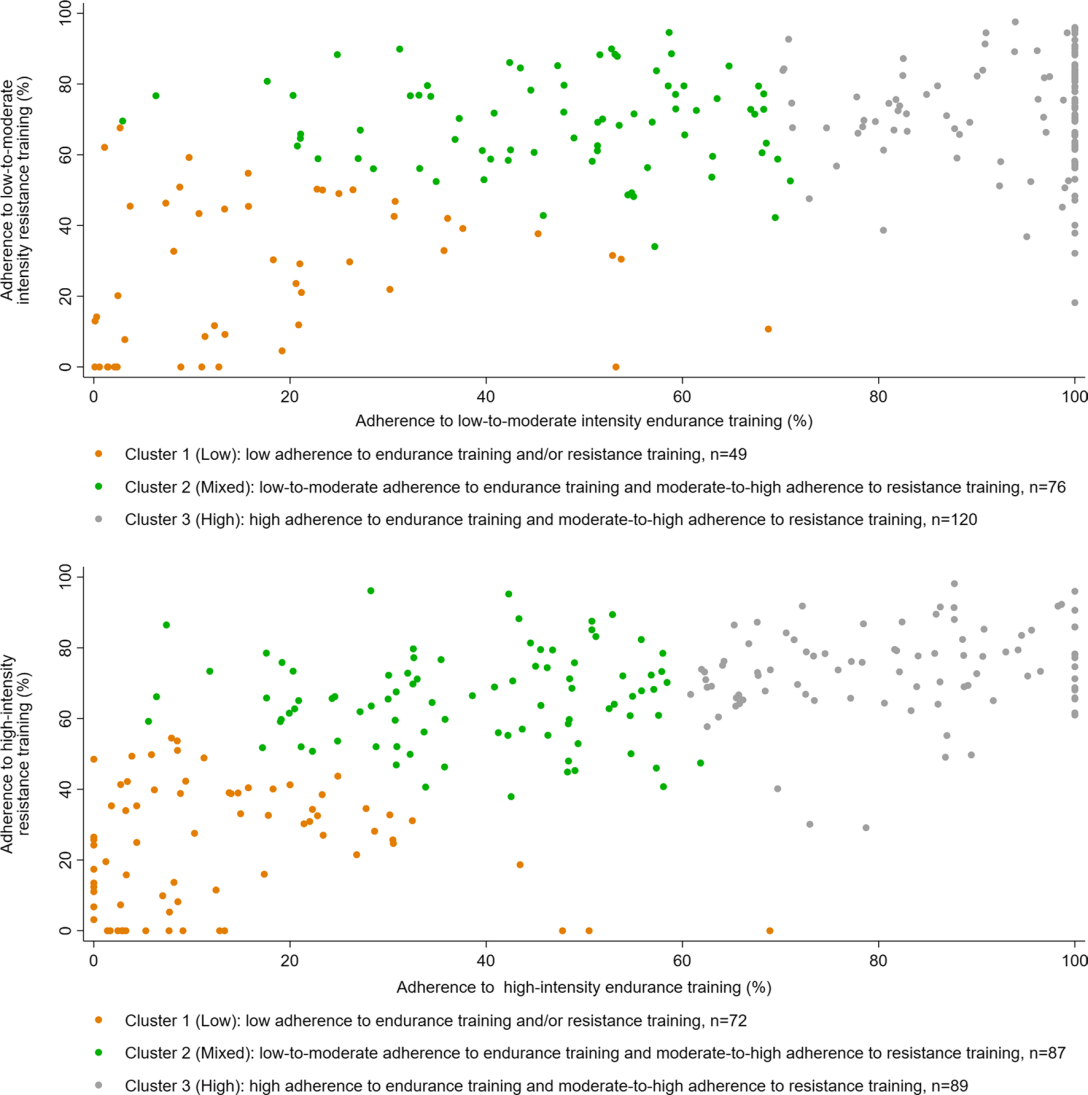
Adherence clusters based on overall adherence within each training intensity group. Orange points represent cluster 1, i.e. individuals within the low adherence to endurance training and/or resistance training; green points represent cluster 2, i.e. individuals with low-to-moderate adherence to endurance training and moderate-to-high adherence to resistance training; grey points represent cluster 3, i.e. individuals with high adherence to endurance training and moderate-to-high adherence to resistance training

For both the low-to-moderate intensity training groups and the high intensity training groups it was most appropriate to divide participants into 3 clusters with similar overall adherence characteristics (Fig. 5).

Individuals in Cluster 1 (Low) had low adherence to endurance training and/or low adherence to resistance training (Table 3). Individuals in Cluster 2 (Mixed) had low-to-moderate adherence to endurance training and moderate-to-high adherence to resistance training (Table 3). Individuals in Cluster 3 (High) had high adherence to endurance training and moderate-to-high adherence to resistance training (Table 3).

**Table 3:** Adherence characteristics of the three identified overall adherence clusters

	Participants randomised to low-to-moderate intensity training (n = 245)		Participants randomised to high intensity training (n = 248)	
	Adherence to endurance training (%)	Adherence to resistance training (%)	Adherence to endurance training (%)	Adherence to resistance training (%)
Cluster 1 (low)	13.4 (3.7–26.1)	29.7 (8.6–45.4)	8.6 (2.9–20.7)	26.1 (7.8–38.8)
Cluster 2 (mixed)	51.3 (35.9–59.1)	69.8 (59.2–78.8)	41.2 (28.3–49.4)	65.7 (56.0–73.4)
Cluster 3 (high)	100 (88.8–100)	73.2 (62.7–82.2)	83.3 (69.7–93.0)	73.8 (66.7–81.2)

Data are median (inter quartile range)

### Potential determinants of membership to adherence clusters

Among participants randomised to low-to-moderate intensity training and among participants randomised to high intensity training, those with higher age, those who were randomised to not receive behaviour change support, those with higher cardiorespiratory fitness at baseline, those with higher scores for fatigue (reduced activity subscale of MFI questionnaire), or higher scores for global health status/quality of life (EORTC C30 subscale) at baseline were less likely to be in Cluster 1 (Low) than Cluster 3 (High) (Table 4).

**Table 4:** Baseline determinants of membership to the three identified overall adherence clusters

	Randomised to low-moderate intensity training								Randomised to high intensity training							
	Low (n = 33) versus high adherence (n = 88) clusters				Mixed (n = 54) versus high adherence (n = 88) clusters				Low (n = 48) versus high adherence (n = 69) clusters				Mixed (n = 58) versus high adherence (n = 69) clusters			
	RRR	LCI	UCI	*P* value	RRR	LCI	UCI	*P* value	RRR	LCI	UCI	*P* value	RRR	LCI	UCI	*P* value
Age	**0.86**	**0.77**	**0.96**	**0.008**	1.01	0.94	1.08	0.877	**0.83**	**0.74**	**0.93**	**0.001**	0.94	0.87	1.02	0.115
Breast cancer	1.00	1.00	1.00		1.00	1.00	1.00		1.00	1.00	1.00		1.00	1.00	1.00	
Colorectal cancer	2.18	0.07	67.2	0.657	**16.9**	**1.37**	**208.2**	**0.027**	0.00	0.00		0.992	5.86	0.12	283.3	0.372
Prostate cancer	1.24	0.07	20.8	0.880	**13.8**	**1.90**	**99.4**	**0.009**	0.59	0.04	8.10	0.691	4.12	0.62	27.2	0.142
Linköping	1.00	1.00	1.00		1.00	1.00	1.00		1.00	1.00	1.00		1.00	1.00	1.00	
Lund	3.99	0.51	31.3	0.188	1.53	0.34	6.93	0.582	**60.4**	**3.60**	**1011.5**	**0.004**	2.17	0.47	9.98	0.321
Uppsala	3.34	0.30	36.7	0.325	0.37	0.08	1.73	0.207	6.38	0.45	90.8	0.172	0.27	0.06	1.19	0.082
with behaviour change support	1.00	1.00	1.00		1.00	1.00	1.00		1.00	1.00	1.00		1.00	1.00	1.00	
without behaviour change support	**0.11**	**0.02**	**0.56**	**0.008**	1.96	0.72	5.31	0.188	**0.20**	**0.05**	**0.85**	**0.029**	1.11	0.40	3.09	0.842
Chemotherapy NO	1.00	1.00	1.00		1.00	1.00	1.00		1.00	1.00	1.00		1.00	1.00	1.00	
Chemotherapy YES	0.89	0.12	6.33	0.905	0.81	0.22	3.02	0.754	0.19	0.03	1.15	0.070	1.25	0.33	4.76	0.739
Comorbidity NO	1.00	1.00	1.00		1.00	1.00	1.00		1.00	1.00	1.00		1.00	1.00	1.00	
Comorbidity YES	1.41	0.36	5.58	0.627	0.84	0.30	2.40	0.751	1.33	0.33	5.37	0.690	1.24	0.44	3.52	0.683
VO_2_ max	**0.81**	**0.69**	**0.94**	**0.006**	0.97	0.89	1.07	0.582	**0.80**	**0.69**	**0.92**	**0.001**	0.91	0.83	1.01	0.067
Endurance training maintenance NO	1.00	1.00	1.00		1.00	1.00	1.00		1.00	1.00	1.00		1.00	1.00	1.00	
Endurance training maintenance YES	0.17	0.02	1.25	0.081	0.41	0.11	1.49	0.177	0.26	0.04	1.82	0.174	0.74	0.21	2.59	0.639
Strength training maintenance NO	1.00	1.00	1.00		1.00	1.00	1.00		1.00	1.00	1.00		1.00	1.00	1.00	
Strength training maintenance YES	7.00	0.52	94.3	0.143	0.25	0.04	1.41	0.115	1.64	0.11	25.51	0.724	1.47	0.36	6.01	0.589
MFI general fatigue	1.15	0.84	1.58	0.376	1.01	0.80	1.27	0.938	0.68	0.45	1.01	0.058	1.13	0.88	1.44	0.338
MFI physical fatigue	0.99	0.77	1.28	0.955	0.80	0.63	1.00	0.054	**1.94**	**1.23**	**3.05**	**0.004**	0.94	0.74	1.21	0.643
MFI reduced action	**0.48**	**0.31**	**0.73**	**0.001**	**0.76**	**0.58**	**1.00**	**0.046**	**0.69**	**0.52**	**0.93**	**0.014**	0.93	0.74	1.16	0.506
MFI reduced motivation	1.30	0.95	1.78	0.099	**1.28**	**1.01**	**1.63**	**0.042**	0.86	0.65	1.13	0.279	0.95	0.77	1.18	0.670
MFI mental fatigue	1.31	0.95	1.79	0.100	**1.36**	**1.08**	**1.72**	**0.010**	1.16	0.87	1.55	0.323	1.13	0.90	1.43	0.304
Global health status/quality of life	**0.95**	**0.90**	**1.00**	**0.041**	**0.94**	**0.91**	**0.99**	**0.009**	**0.93**	**0.88**	**0.98**	**0.007**	0.99	0.95	1.03	0.728
Physical function	1.01	0.93	1.09	0.787	0.99	0.93	1.04	0.603	0.97	0.90	1.04	0.388	1.01	0.95	1.06	0.802
Role function	**1.05**	**1.00**	**1.09**	**0.036**	1.01	0.98	1.04	0.392	1.02	0.99	1.06	0.176	1.02	1.00	1.05	0.069
Emotional function	1.00	0.94	1.05	0.912	0.99	0.96	1.03	0.730	1.00	0.95	1.06	0.997	**1.05**	**1.01**	**1.09**	**0.022**
Cognitive function	1.01	0.95	1.08	0.700	**1.05**	**1.01**	**1.09**	**0.007**	**1.07**	**1.01**	**1.14**	**0.028**	1.02	0.98	1.06	0.397
Social function	1.01	0.97	1.05	0.691	1.02	0.98	1.06	0.365	1.02	0.97	1.08	0.406	0.96	0.93	1.00	0.035
Nausea/vomiting	1.03	0.93	1.13	0.600	1.03	0.96	1.10	0.450	1.06	0.96	1.16	0.225	0.99	0.92	1.06	0.782
Pain	0.99	0.95	1.04	0.732	1.02	0.98	1.05	0.330	0.96	0.92	1.00	0.073	1.00	0.97	1.03	0.847
Dyspnoea	1.00	0.94	1.05	0.952	0.99	0.95	1.04	0.749	0.99	0.95	1.03	0.675	0.99	0.95	1.03	0.516
Insomnia	0.98	0.95	1.01	0.165	0.99	0.97	1.01	0.478	1.02	0.99	1.05	0.253	1.00	0.98	1.03	0.902
Appetite loss	0.99	0.96	1.03	0.741	1.01	0.98	1.04	0.526	1.04	0.99	1.09	0.100	1.00	0.97	1.04	0.826
Constipation	**1.07**	**1.02**	**1.13**	**0.005**	**1.04**	**1.01**	**1.08**	**0.026**	0.99	0.95	1.04	0.691	0.99	0.95	1.03	0.533
Diarrhoea	0.98	0.94	1.03	0.419	1.00	0.97	1.03	0.885	1.02	0.99	1.06	0.196	1.00	0.98	1.04	0.746

Main findings are highlighted in bold text

Three clusters identified: Low adherence cluster (low adherence to endurance training and/or low adherence to resistance training), Mixed adherence cluster (low-to-moderate adherence to endurance training and moderate-to-high adherence to resistance training) and High adherence cluster (individuals with high adherence (high adherence to endurance training and moderate-to-high adherence to resistance training)

RRR, relative risk ratio; LCI, lower confidence interval; UCI, upper confidence interval; MFI, multidimensional fatigue inventory

Among participants randomised to low-to-moderate intensity training, those with higher scores for fatigue (reduced activity subscale of MFI questionnaire) or global health status/quality of life were also less likely to be in Cluster 2 (Mixed) than Cluster 3 (High) (Table 4). In contrast, those with higher scores for role function and constipation (EORTC C30 subscales) were more likely to be in Cluster 1 (Low) than Cluster 3 (High), and those with colorectal or prostate cancer, with higher scores for reduced motivation or mental fatigue (MFI subscales), or with higher scores for cognitive function or constipation (EORTC C30 subscale) were more likely to be in Cluster 2 (Mixed) than Cluster 3 (High).

Among participants randomised to high intensity training, those with higher scores for physical fatigue (MFI subscale) or higher scores for cognitive function (EORTC C30 subscale) were more likely to be in Cluster 1 (Low) than Cluster 3 (High) and those with higher scores for emotional function (MFI subscale) were more likely to be in Cluster 2 (Mixed) than Cluster 3 (High) (Table 4).

## Discussion

Among participants randomised to low-to-moderate intensity training and those randomised to high intensity training, we found a positive curvilinear correlation between adherence to resistance training and endurance training overall. This was also seen for training frequency among participants randomised to high intensity training. Among participants randomised to high intensity training, adherence to intensity and time for resistance training was consistently high, while for endurance training there were many different combinations of adherence to each of the four FITT principles. Age, randomisation group with regards to behaviour change support, and baseline cardiorespiratory fitness, fatigue (reduced activity subscale of MFI questionnaire) and quality of life score were the key determinants of membership to adherence clusters among participants randomised to low-to-moderate intensity training and among participants randomised to high intensity training. Among participants randomised to low-to-moderate intensity training, role function score, symptoms of constipation, cancer type, reduced motivation score, mental fatigue score and cognitive function score were additional determinants of membership to adherence clusters. Further determinants of membership to adherence clusters among participants randomised to high intensity training were physical fatigue score, cognitive function score, and emotional function score.

Among participants with low adherence to individual home-based endurance training, adherence was higher for the supervised group-based resistance training. This was true among participants randomised to low-to-moderate intensity training and those randomised to high intensity training. The importance of the structure, scheduling and social support provided by group-based exercise during and after treatment for cancer has previously been described [16–18], and may explain this result. Earlier studies in patients undergoing cancer treatment have reported a mean attendance of 60–70% when endurance training sessions have been supervised [19, 20]. This suggests that challenges to adherence for endurance training may have been reduced in a supervised setting. Despite this, the plateau in adherence to resistance training around 65–75% may point to a disadvantage of the scheduled nature of the supervised resistance training sessions. Participants who missed a resistance training session may have lacked motivation or ability to perform the session at another time during their demanding cancer treatment, since the ‘compensation’ session would be self-initiated and not supervised or group-based.

The structured and guided environment of the resistance training may also explain the consistently high levels of adherence to intensity, time, and to a large extent, type in the participants randomised to high intensity training. Among participants randomised to high intensity training, there were many more combinations of adherence levels for the four FITT principles for endurance training compared to resistance training. This suggests that there were challenges to adhere to each of the FITT principles for endurance training. For example, it may have been challenging to adhere to the frequency principle for endurance training, since participants needed to motivate themselves to perform the exercise sessions alone in their home environment. Further, since there was no supervision or guidance during the sessions it was not possible to ensure the interval training was performed according to the protocol, this could have impacted intensity, time and type. In addition, and participants may have felt uncomfortable pushing themselves to the high intensity prescribed, so may have chosen to exercise at a lower intensity or for longer or shorter intervals according to their preference.

There were not substantial groups of participants with high adherence to resistance training but low adherence to endurance training or vice versa. This suggests that adherence can be considered an overarching correlated behavioural pattern within individuals that is typically not distorted by preference for one particular type of training. Nonetheless, among participants randomised to high intensity training, adherence to exercise type was the one FITT principle where there was a subset of participants with very low adherence for resistance training that was not seen in the endurance training. These participants often had high adherence to frequency, intensity and time. This observation is likely to reflect physical barriers to performing the prescribed exercises at the gym. In these situations the exercises were adapted to the participants’ limitations in accordance with the intervention protocol. For example, patients with breast surgery may have struggled to perform some of the upper body resistance exercise at the correct intensity, so they were instructed to perform either a similar exercise (i.e. same muscle group working) at a lower intensity (e.g. using resistance bands or merely body weight), or another exercise involving other upper body muscle groups (e.g. triceps dips).

Our results suggest that in future interventions, participants with lower age, lower fitness levels, less fatigue, and lower quality of life, may require additional strategies to encourage adherence to exercise prescriptions of both low-to-moderate and high intensity. Our results are in line with previous studies that have shown higher age [21] and baseline fitness [21–24] to be associated with higher adherence to exercise intervention during treatment in cancer survivors. However, in contrast to our findings, others have shown inverse associations or have not found associations of age [22, 23, 25, 26], baseline fitness level [26, 27] and quality of life [23, 26] with adherence to exercise interventions during cancer treatment. These mixed results suggest that determinants of adherence to exercise interventions may be dependent on the content and context of the intervention, since there has been wide variety in the type, intensity and duration of exercise interventions that have been delivered, and many different populations and cancer types that have been previously studied. The association of higher physical fatigue with lower adherence has also been reported in some [22], but not all [23, 25, 26] previous studies. It was interesting that physical fatigue was a determinant of intervention adherence among participants randomised to high-intensity training, but not among those randomised to low-to-moderate intensity training. This suggests that participants with higher levels of baseline fatigue may require extra support to achieve high levels of adherence to exercise interventions if they are required to train at high intensity, that may not be required if the training prescribed is of low-to-moderate intensity. No particular treatment or disease related symptoms, except constipation, were associated with adherence to the exercise interventions, even though disease and treatment-related symptoms have previously been suggested as reasons for reduced attendance of exercise interventions during treatment [28]. Nonetheless, symptoms such as fatigue are known to vary across chemotherapy cycles [29], so it may be that measuring baseline symptoms does not capture the relevant timeframe for these potential determinants of adherence.

The main strength of this study is the detailed information about adherence to resistance and endurance training acquired from a large, rigorously conducted, multicentre RCT, which allowed adherence to resistance and endurance training to be examined in parallel. One limitation was that it was not possible to compare resistance and endurance training adherence for individual FITT principles for the low-to-moderate intensity training groups, since training frequency was not stipulated in the low-to-moderate intensity training protocol. Further, our results may have limited generalisability since, as in all exercise-trials, participants were a subgroup of the population who were willing to participate in a 6 month exercise intervention during treatment for cancer, and they were largely highly educated and already planning to become physically active at baseline.

## Conclusions

Our results suggest that (1) in exercise interventions during cancer treatment that include both endurance training and resistance training, adherence can be considered an overarching correlated behavioural pattern within individuals, that is typically not distorted by preference for one particular type (resistance vs. endurance) of training, (2) around 10% patients undergoing treatment for cancer may not be able to perform the *type* of high intensity resistance exercises at the gym that has been prescribed, even if they are able to adhere well to the prescribed *frequency*, *intensity* and *time* of these exercises—future interventions may need to be personalised to take this into account; (3) in future interventions additional support encouraging adherence to exercise prescriptions may be beneficial for participants with lower age, lower fitness levels, less fatigue, and lower quality of life at the start of their cancer treatment, for both low-to-moderate and high intensity exercise interventions, while participants with higher levels of baseline fatigue may require extra support to achieve high levels of adherence to exercise interventions if they are required to train at high intensity.

## Data Availability

De-identified participant data (including data dictionaries) will be shared upon reasonable request for research purposes by contacting the corresponding author.

## Abbreviations

HI: High intensity training
LMI: Low-to-moderate intensity training
FITT: Frequency, intensity, time, and type
RM: Repetition maximum
MFI: Multidimensional Fatigue Inventory
EORTC: European Organization for Research and Treatment of Cancer
QLQ-C30: Quality of Life Questionnaire

## References

[1] Campbell KL, Winters-Stone KM, Wiskemann J, May AM, Schwartz AL, Courneya KS (2019). Exercise guidelines for cancer survivors: consensus statement from international multidisciplinary roundtable. Med Sci Sports Exerc.

[2] Meneses-Echavez JF, Gonzalez-Jimenez E, Ramirez-Velez R (2015). Effects of supervised multimodal exercise interventions on cancer-related fatigue: systematic review and meta-analysis of randomized controlled trials. Biomed Res Int.

[3] Gerritsen JK, Vincent AJ (2016). Exercise improves quality of life in patients with cancer: a systematic review and meta-analysis of randomised controlled trials. Br J Sports Med.

[4] Neil-Sztramko SE, Medysky ME, Campbell KL, Bland KA, Winters-Stone KM (2019). Attention to the principles of exercise training in exercise studies on prostate cancer survivors: a systematic review. BMC Cancer.

[5] Ormel HL, van der Schoot GGF, Sluiter WJ, Jalving M, Gietema JA, Walenkamp AME (2018). Predictors of adherence to exercise interventions during and after cancer treatment: a systematic review. Psychooncology.

[6] Neil-Sztramko SE, Winters-Stone KM, Bland KA, Campbell KL (2019). Updated systematic review of exercise studies in breast cancer survivors: attention to the principles of exercise training. Br J Sports Med.

[7] Mazzoni AS, Brooke HL, Berntsen S, Nordin K, Demmelmaier I (2020). Exercise adherence and effect of self-regulatory behavior change techniques in patients undergoing curative cancer treatment: secondary analysis from the phys-can randomized controlled trial. Integr Cancer Ther.

[8] Kirkham AA, Bonsignore A, Bland KA, McKenzie DC, Gelmon KA (2018). Exercise prescription and adherence for breast cancer: one size does not FITT all. Med Sci Sports Exerc..

[9] Kampshoff CS, Jansen F, van Mechelen W, May AM, Brug J, Chinapaw MJ (2014). Determinants of exercise adherence and maintenance among cancer survivors: a systematic review. Int J Behav Nutr Phys Act.

[10] Berntsen S, Aaronson NK, Buffart L, Borjeson S, Demmelmaier I, Hellbom M (2017). Design of a randomized controlled trial of physical training and cancer (Phys-Can)—the impact of exercise intensity on cancer related fatigue, quality of life and disease outcome. BMC Cancer.

[11] Demmelmaier I, Brooke HL, Henriksson A, Mazzoni AS, Bjorke ACH, Igelstrom H (2021). Does exercise intensity matter for fatigue during (neo-)adjuvant cancer treatment? The Phys-Can randomized clinical trial. Scand J Med Sci Sports.

[12] Edvardsen E, Hansen BH, Holme IM, Dyrstad SM, Anderssen SA (2013). Reference values for cardiorespiratory response and fitness on the treadmill in a 20- to 85-year-old population. Chest.

[13] Smets EM, Garssen B, Bonke B, De Haes JC (1995). The Multidimensional Fatigue Inventory (MFI) psychometric qualities of an instrument to assess fatigue. J Psychosom Res.

[14] Aaronson NK, Ahmedzai S, Bergman B, Bullinger M, Cull A, Duez NJ (1993). The European Organization for Research and Treatment of Cancer QLQ-C30: a quality-of-life instrument for use in international clinical trials in oncology. J Natl Cancer Inst.

[15] Makles A (2012). Stata tip 110: How to get the optimal k-means cluster solution. Stata J.

[16] Mazzoni AS, Carlsson M, Berntsen S, Nordin K, Demmelmaier I (2019). "Finding my own motivation"—a mixed methods study of exercise and behaviour change support during oncological treatment. Int J Behav Med.

[17] Midtgaard J, Hammer NM, Andersen C, Larsen A, Bruun DM, Jarden M (2015). Cancer survivors' experience of exercise-based cancer rehabilitation—a meta-synthesis of qualitative research. Acta Oncol.

[18] Browall M, Mijwel S, Rundqvist H, Wengstrom Y (2018). Physical activity during and after adjuvant treatment for breast cancer: an integrative review of women's experiences. Integr Cancer Ther.

[19] Hwang CL, Yu CJ, Shih JY, Yang PC, Wu YT (2012). Effects of exercise training on exercise capacity in patients with non-small cell lung cancer receiving targeted therapy. Support Care Cancer.

[20] Mijwel S, Backman M, Bolam KA, Jervaeus A, Sundberg CJ, Margolin S (2018). Adding high-intensity interval training to conventional training modalities: optimizing health-related outcomes during chemotherapy for breast cancer: the OptiTrain randomized controlled trial. Breast Cancer Res Treat.

[21] Arem H, Sorkin M, Cartmel B, Fiellin M, Capozza S, Harrigan M (2016). Exercise adherence in a randomized trial of exercise on aromatase inhibitor arthralgias in breast cancer survivors: the Hormones and Physical Exercise (HOPE) study. J Cancer Survivorship Res Practice.

[22] Shang J, Wenzel J, Krumm S, Griffith K, Stewart K (2012). Who will drop out and who will drop in exercise adherence in a randomized clinical trial among patients receiving active cancer treatment. Cancer Nurs.

[23] Courneya KS, Segal RJ, Gelmon K, Reid RD, Mackey JR, Friedenreich CM (2008). Predictors of supervised exercise adherence during breast cancer chemotherapy. Med Sci Sports Exerc.

[24] Courneya KS, Segal RJ, Gelmon K, Mackey JR, Friedenreich CM, Yasui Y (2014). Predictors of adherence to different types and doses of supervised exercise during breast cancer chemotherapy. Int J Behav Nutr Phys Act.

[25] Swenson KK, Nissen MJ, Henly SJ (2010). Physical activity in women receiving chemotherapy for breast cancer: adherence to a walking intervention. Oncol Nurs Forum.

[26] Courneya KS, Segal RJ, Reid RD, Jones LW, Malone SC, Venner PM (2004). Three independent factors predicted adherence in a randomized controlled trial of resistance exercise training among prostate cancer survivors. J Clin Epidemiol.

[27] Courneya KS, Friedenreich CM, Quinney HA, Fields AL, Jones LW, Fairey AS (2004). Predictors of adherence and contamination in a randomized trial of exercise in colorectal cancer survivors. Psychooncology.

[28] Courneya KS, Friedenreich CM, Quinney HA, Fields AL, Jones LW, Vallance JK (2005). A longitudinal study of exercise barriers in colorectal cancer survivors participating in a randomized controlled trial. Ann Behav Med.

[29] Kirkham AA, Bland KA, Zucker DS, Bovard J, Shenkier T, McKenzie DC (2020). "Chemotherapy-periodized" exercise to accommodate for cyclical variation in fatigue. Med Sci Sports Exerc.

